# Speciation and hybridization in invasive fire ants

**DOI:** 10.1186/s12862-019-1437-9

**Published:** 2019-05-29

**Authors:** Pnina Cohen, Eyal Privman

**Affiliations:** Department of Evolution and Environmental Biology, Institute of Evolution, University of Haifa, Haifa, Israel

## Abstract

**Background:**

A major focus of evolutionary biology is the formation of reproductive barriers leading to divergence and ultimately, speciation. Often, it is not clear whether the separation of populations is complete or if there still is ongoing gene flow in the form of rare cases of admixture, known as isolation with migration. Here, we studied the speciation of two fire ant species, *Solenopsis invicta* and *Solenopsis richteri*, both native to South America, both inadvertently introduced to North America in the early twentieth century. While the two species are known to admix in the introduced range, in the native range no hybrids were found.

**Results:**

We conducted a population genomic survey of native and introduced populations of the two species using reduced representation genomic sequencing of 337 samples. Using maximum likelihood analysis over native range samples, we found no evidence of any gene flow between the species since they diverged. We estimated their time of divergence to 190,000 (100,000–350,000) generations ago. Modelling the demographic history of native and introduced *S. invicta* populations, we evaluated their divergence times and historic and contemporary population sizes, including the original founder population in North America, which was estimated at 26 (10–93) unrelated singly-mated queens.

**Conclusions:**

We provide evidence for complete genetic isolation maintained between two invasive species in their natïve range, based, for the first time, on large scale genomic data analysis. The results lay the foundations for further studies into different stages in the formation of genetic barriers in dynamic, invasive populations.

**Electronic supplementary material:**

The online version of this article (10.1186/s12862-019-1437-9) contains supplementary material, which is available to authorized users.

## Background

The separation of populations and their eventual speciation is a fundamental process and a driving force in evolution. However, a newly formed reproductive barrier is often incomplete. Hybridization and introgression between incipient species, or gene flow from one species’ gene pool into another by backcrossing, may still occur. Many cases of species hybridization, resulting from accidental introduction of foreign species into new environments, have been identified and documented [1–3]. Often the outcome of human activities, these systems provide an opportunity to dissect the mechanisms underlying the formation of reproductive barriers. Exploring the differences between populations and their environments in native and introduced ranges may reveal the factors responsible for genetic isolation, which would eventually result in irreversible speciation.

Here, we describe one such case study involving two invasive fire ant species. These species, thought to maintain genetic isolation in their native range [4–6], freely hybridize in their newly introduced range [7, 8]. Unlike other incidents of introduction resulting in admixture, the two species are parapatric in both the native and the introduced ranges. Furthermore, the fact that the two ranges are located thousands of kilometers apart, allows for clear distinction between the native and introduced populations under study. This makes it a unique system which can help to provide insights into the evolution of genetic isolation and speciation.

The red fire ant, *Solenopsis invicta*, and the black fire ant, *S. richteri*, are closely related species, native to South America. Both species were inadvertently introduced to North America in the early twentieth century [9–11], with *S. invicta* subsequently migrating to other countries worldwide [12, 13]. Since they were first detected in the USA, the fire ant species have been closely monitored and their spread across the southeast is well documented. Their life cycle, behavior, genetic makeup, population structure and invasion history are the subject of many publications, making these species, in particular *S. invicta*, amongst the most studied invasive species, and an excellent subject for demographic history analysis.

*S. invicta* and *S. richteri*’ display social polymorphism, with two distinct social colony structures – the monogyne (single queen) and the polygyne (multiple queens) forms. The social polymorphism is a Mendelian trait determined by a supergene of 13 megabases, and is marked by the gene *gp-9* with monogyne queens always having *gp-9*^*BB*^ genotypes and polygyne queens always having *gp-9*^*Bb*^ genotypes [14–16]. The social structure of a colony greatly affects its size, longevity, and dispersion patterns [17, 18].

Population genetics analysis of microsatellite and mitochondrial genotypes of thousands of *S. invicta* colonies around the world were used to trace the place of origin of the introduced *S. invicta* populations to the Formosa region in northern Argentina [11, 13]. It was also established that all subsequent introductions of *S. invicta* across the globe originated in the introduced USA population. The population of origin of the introduced *S. richteri* is still unknown.

The number of *S. invicta* queens that were initially introduced to North America was estimated through the screening of haplotypes in mtDNA sequences, as well as genotypes of allozymes, microsatellites, and the complementary sex determination (CSD) locus in individuals sampled throughout the introduced range [19]. It was determined that to account for all of the allele variants, the original group that was introduced to USA consisted of 9–30 unrelated mated queens.

In the introduced range, admixture between *S. invicta* and *S. richteri* is prevalent [7, 8]. A hybrid zone was identified ranging from Georgia in the east through Alabama to central Mississippi [18, 20, 21]. This is not the case in the native range of South America, where admixture was found to be a rare occurrence [4], or not evident at all [4–6]. While extensive sampling of native colonies was conducted, no more than 26 nuclear genetic markers were used in testing for admixture between the species, which limits the power of the analysis to detect gene flow.

We used large scale population genomic data to study the demographic history of *S. invicta* and *S. richteri*. Our data include samples from both species in both native and introduced ranges. The hybrid zone was not included in the sampling because the admixture in the introduced range has been well established in previous studies [7, 8] . These genomic data allow for an explicit maximum likelihood test for historic gene flow between the *S. invicta* and *S. richteri* in their native range to determine if these species had indeed maintained genetic isolation since their separation. We also provide estimates for speciation times, population divergence times and historic and contemporary effective population sizes, including the founding *S. invicta* population in North America. This is the first demographic history study of these species that uses thousands of genomic markers, which allow population genetic model inference at high accuracy and provide the necessary statistical power to test for gene flow.

## Results

**Fig. 1:**
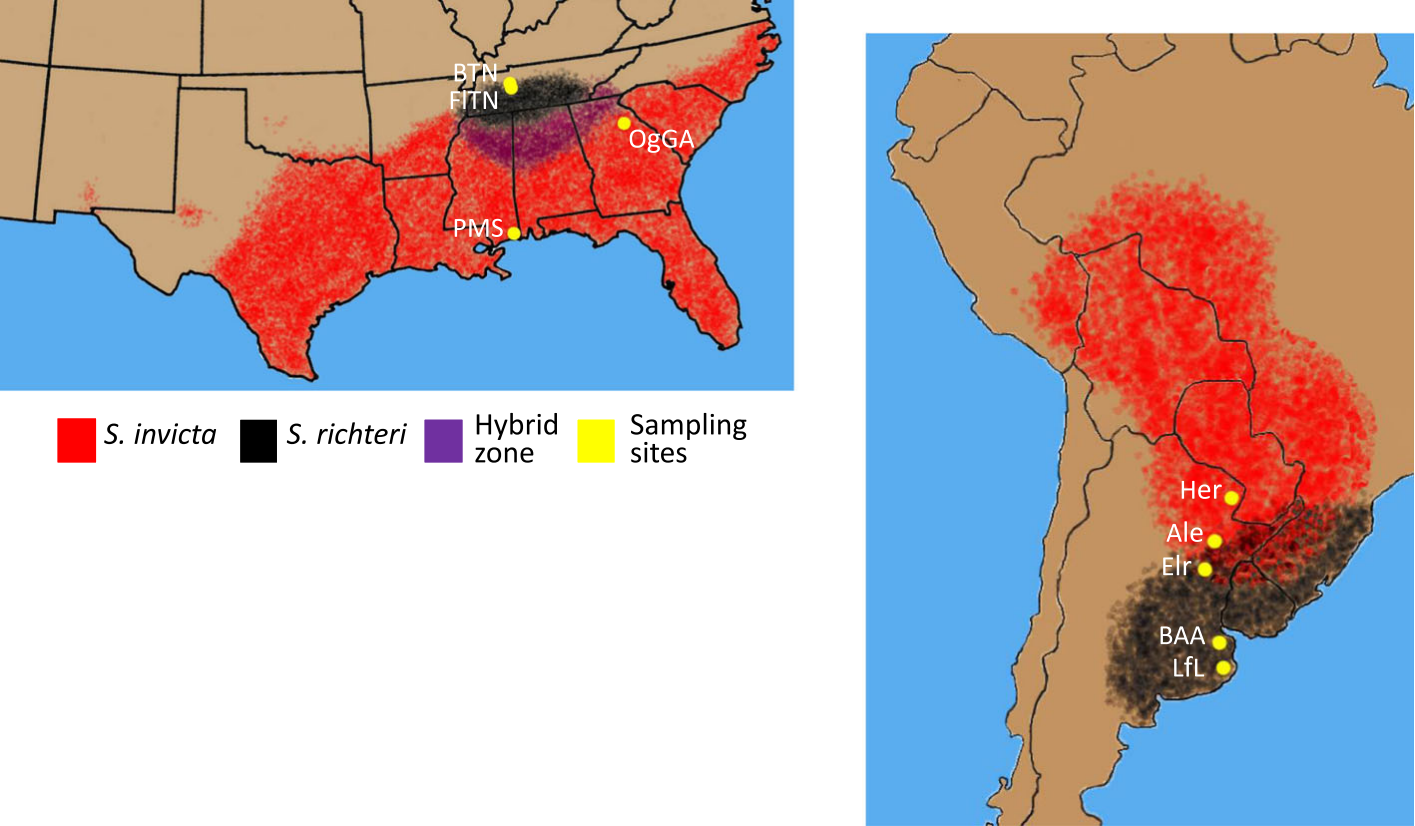
Distribution map of *S. invicta* and *S. richteri* across North and South America with marked sampling sites. *S. invicta* was sampled in three sites in its native range (*Her*, *Ale*, *Elr*) and in two sites in the introduced range (*OgGA*, *PMS*). *S. richteri* was sampled in two sites in its native range (*BAA*, *LfL*) and in two sites in the introduced range (*BTN*, *FlNT*). The area marked in purple shows the hybridization zone between *S. invicta* and *S. richteri.* Distribution is based on [13, 60–63] and https://www.aphis.usda.gov/plant_health/plant_pest_info/fireants/downloads/fireant.pdf. Sampled populations are: *Herr* - Herradura, *Elr* – El Recreo, *Ale* – Alejandra, *PMS* - Pascagoula, Mississippi, *OgGA* - Oglethorpe Co., Georgia, *BAA* - Buenos Aires, *LfL* - Las Flores, *BTN* - Benton Co., Tennessee *FlTN* - Flatwood, Tennessee

We inferred the population structure and demographic history using RAD sequencing of population samples of *S. invicta* and *S. richteri* from nine localities in their native range in Argentina and their introduced range in the USA (Fig. 1; Additional file 1: Tables S1 and S2). Altogether, this dataset consists of 962,896,602 sequenced reads, 96 nucleotides long each, from 337 samples. After stringent quality filtering, the genotypes of between 6389 and 285,847 SNPs, representing between 161 and 337 individuals (depending on filtering parameters and analysis type) and a total of 16,648 aligned RADseqs were used in the different analyses (see Methods).

### Population structure

**Fig. 2:**
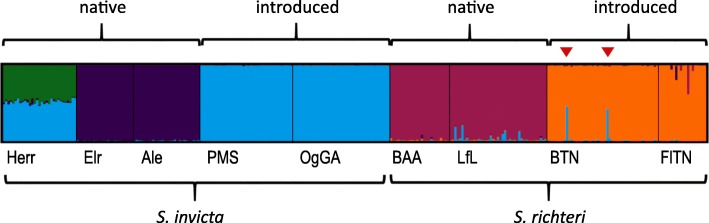
Structure analysis of the studied populations. Summary of 76 of 100 runs of *STRUCTURE* with *K* = 5. Each individual is shown as a vertical bar, colored in accordance to its inferred clustering. Individuals of eight of the nine populations are clustered according to species and range, with the exception of two individuals marked in red triangles. These are mapped to both introduced *S. invicta* and *S. richteri* clusters, probably due to hybrid ancestry. Individuals of Herradura population are mapped to a fifth cluster, and to a cluster shared with the introduced *S. invicta* ants

*STRUCTURE* [22, 23] is a Bayesian clustering method that assigns individuals to ancestry clusters. *STRUCTURE* analysis resulted in the identification of five distinct population clusters (Fig. 2). No further substructure was found for *K* > 5 (Additional file 1: Figure S1). In 76 of 100 *STRUCTURE* runs with *K* = 5, the four introduced populations were clustered into two separate clusters, one for each species. Two individuals of the introduced *S. richteri* population of Benton, Tennessee, had inferred ancestry belonging to both clusters, indicating possible hybrids. The two native *S. richteri* populations were assigned to a single cluster, while two of the native *S. invicta* populations (Alejandra and El Recreo) were assigned to a fourth cluster. Individuals of the third native *S. invicta* population, sampled in Herradura, were partially assigned to the cluster of the introduced *S. invicta* populations and partially to a separate, fifth cluster.

The allocation of populations into clusters suggests that the polymorphism in the introduced *S. invicta* is a subset of the genetic variants found in the native Herradura samples, making the latter a possible population of origin of the former. This result is in line with the previous studies that identified the Formosa region, which includes Herradura, as the likely source of the introduced *S. invicta* [11, 13]. Unlike the pattern observed in the Herradura population, neither of the sampled native *S. richteri* populations share a cluster with the introduced populations, indicating that these are not the source population of the introduced *S. richteri*.

We calculated the genetic distances between each pair of populations, using the genetic differentiation measure of *F*_*ST*_. The results can be found in Additional file 1: Table S3.

### Reproductive isolation in the native range

We tested for historic and contemporary gene flow between the native *S. invicta* and *S. richteri* populations by comparing between two competing demographic history models. To this end, we used a full maximum likelihood analysis of coalescent models [24, 25] as implemented in the *3s* software [26]. Based on the phylogenetic inference, the maximum likelihood scores of two possible demographic history models are compared. A null model that does not allow genetic flow between the two closely related species and an alternative model that does.

This analysis concluded with an insignificant difference between the likelihood scores of the two scenarios (*ΔlnL* = 0.06; *p* > 0.9). Consequently, the null model could not be rejected, indicating that there was no evidence of gene flow between the native *S. invicta* and *S. richteri* populations since their divergence from a common ancestral population.

### Speciation times and population sizes

**Fig. 3:**
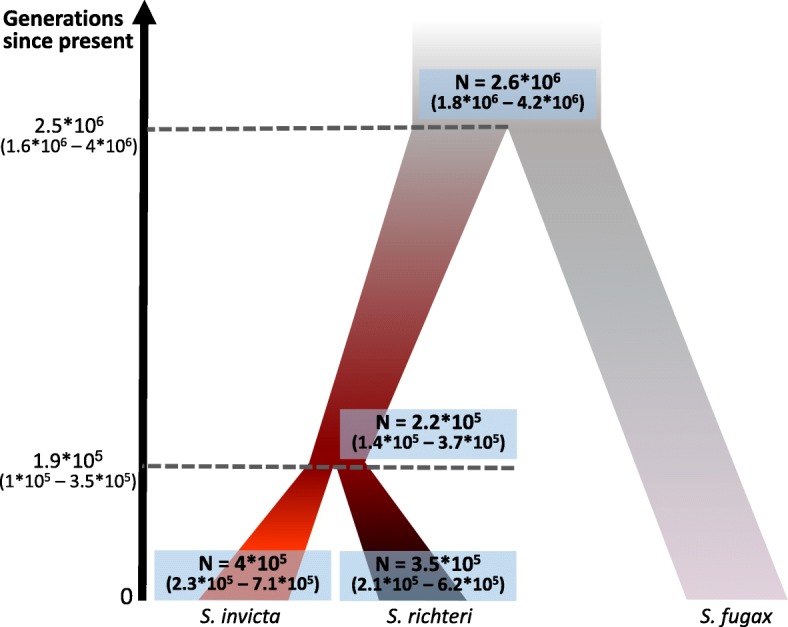
Population effective sizes and generations number since the two speciation events. The values were calculated using the mutation rate of 3.4*10^− 9^ with the 95% confidence level range of 2.2*10^− 9^ - 4.9*10^− 9^ over *3S*’s estimates (Table 1). Marked in blue are the population effective sizes and to the left is the time line in generations number

*3s* provided maximum likelihood estimates for the null model parameters, including effective population sizes and the time since the speciation of *S. invicta* and *S. richteri*. Since we used the sequence of an individual of a different *Solenopsis* species, *Solenopsis fugax*, as an out-group, the model provided an estimate to the speciation time of this species from the lineage leading to *S. invicta* and *S. richteri*. Estimates were also provided to the effective size of the populations of *S. richteri* and *S. invicta*, the effective size of the ancestral population of *S. invicta* and *S. richteri* and that of the ancestral population of all three *Solenopsis* species (Table 1). Using the mutation rate of 3.4*10^− 9^ bp/generation (95% confidence interval of 2.2*10^− 9^ – 4.9*10^− 9^) that was estimated for the honey bee [28, 29], we calculated that the speciation of *S. invicta* and *S. richteri* happened 1.9*10^5^ (1*10^5^–3.5*10^5^) generations ago and the speciation of *S. fugax* from the lineage leading to *S. invicta* and *S. richteri* took place 2.5*10^6^ (1.6*10^6^–4*10^6^) generations ago. See Fig. 3 for all estimated parameters.

**Table 1:** *3s* parameter estimates

	Ancestral population to *S. invicta* and *richteri*	Ancestral population to *S*. *invicta*, *richteri* and *fugax*	*S. richteri*	*S. invicta*
Temporal parameters	0.000646 (0.00052–0.00077)	0.008369 (0.00789–0.00884)		
Demographic parameters	0.002293 (0. 00211–0.00247)	0.026874 (0.02591–0.02783)	0.003596 (0.00309–0.00409)	0.004062 (0.00343–0.00469)

*3s* estimates of the null model parameters in the highest scoring run

The estimates for the effective population sizes were similar between present-day populations of *S. invicta* and *S. richteri*, while the ancestral population of these two populations was found to be slightly smaller. The effective size of the ancestral population of the three *Solenopsis* species was found to be larger than the other populations by an order of magnitude, indicating a very diverse ancestral population. See Fig. 3 for the exact numbers.

#### Founder population of introduced *S. invicta*

We estimated the demographic and temporal parameters that define the history of *S. invicta* populations using Approximate Bayesian Computation (ABC) [30], as implemented in *DIYABC* v2.0.4 [31, 32]. Unfortunately, it was not possible to fit a similar model to the *S. richteri* populations (poor goodness of fit). This may be because we did not sample the source population of the *S. richteri* introduction.

**Fig. 4:**
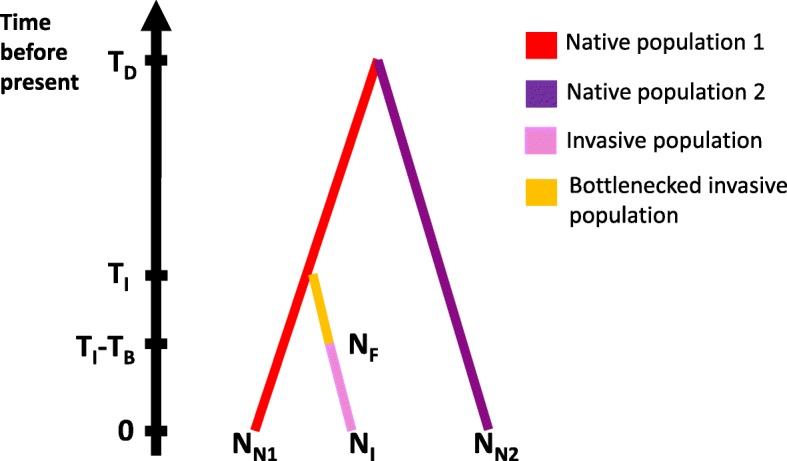
Model of *S. invicta* demographic history. The model describes the population history starting with the divergence within the native populations and followed by the introduction to North America, which originated from the Herradura population. Populations: *N*_*N1*_ – native population of Herradura; *N*_*N2*_ – native population of Alejandra and El Recreo; *N*_*F*_ – bottlenecked founder population in the USA; *N*_*I*_ – contemporary population in the USA. Times: 0 – populations sampling; *T*_*I*_ – introduction; *T*_*B*_ – length of bottleneck for the introduced population; *T*_*D*_ - divergence of the Herradura population from the Alejandra and El Recreo populations

An ABC analysis is often used to compare between multiple competing historic demographic models that differ in their scenarios. As many details in the history of *S. invicta* are already established, an alternative scenario is unnecessary. Rather, we concentrated on estimating the model parameters of population sizes and time of demographic events as depicted in Fig. 4.

With a maximum posterior of 39 (95% credible interval of 14–139), the effective size of the founding population is quite low. The length of the bottleneck period of the introduced *S. invicta* (*T*_*B*_) is minimal and stands at 2 generations for the maximum posterior estimate, with 95% credible interval ranging between 0 and 16 generations. Combined with the small size of the founder population, this suggests a rapid increase in population size immediately following the introduction. It is in line with historic records in which the presence of the newly introduced fire ants was initially reported as a mere curiosity and quickly changed into a growing concern and even panic, as ant numbers exploded [18].

The effective population size of the Herradura population (*N*_*N1*_) is approximately two orders of magnitude larger than the second native population cluster (*N*_*N2*_) estimated at 1.2*10^7^ and 2.5*10^5^, respectively (95% credibility interval of 3.3*10^6^–7.8*10^7^ and 8.2*10^4^–2*10^6^, respectively).

**Fig. 5:**
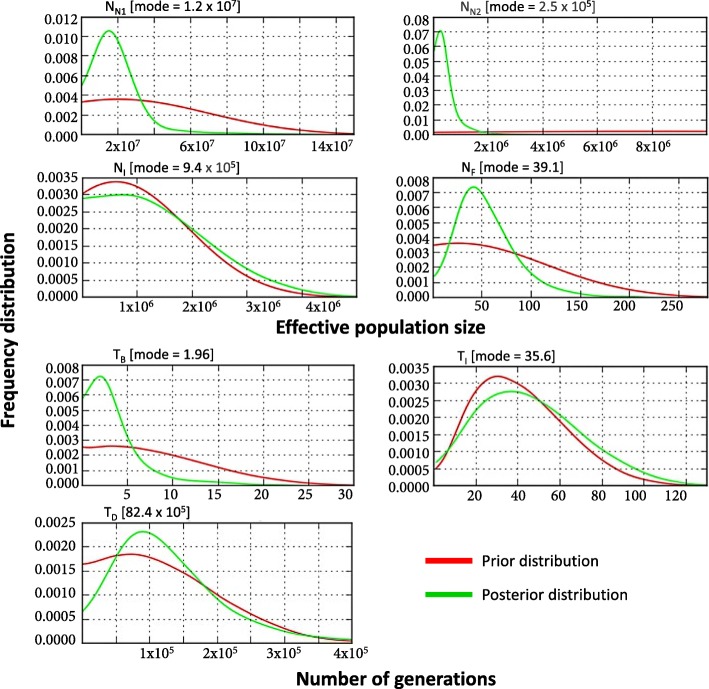
Demographic model parameters inferred for *S. invicta* populations. The prior and posterior density distributions are plotted in red and green, respectively. The maximum posterior estimate of each of the parameters is indicated above its plot. Parameters defined in Fig. 2

In Fig. 5, we plotted the full posterior distributions of all model parameters. The numeric values of these distributions, including their 95% quantiles, are summarized in Additional file 1: Table S4. For parameters *N*_*I*_, *T*_*I*_ and *T*_*D*_, the posterior density distributions are very similar to their prior density distributions, indicating that the analysis could not extract information from the given data. The first two parameters are directly linked to the recent introduction to the USA, and the rapid increase in population size that followed. The sudden shift in dynamics from the originally stabilized population in the native range may have resulted in poor representation of the introduced populations by their summary statistics. Nevertheless, a ‘goodness of fit’ analysis indicated that the observed genetic data are well explained by the model and its parameter posterior distributions (Additional file 1: Figure S4).

## Discussion

We explicitly tested two competing demographic history models and found that the reproductive barrier between two closely related species of fire ants was sustained for about 200,000 generations, and is still preserved in their native range of South America. The *STRUCTURE* analysis of 171 native samples seems to indicate low levels of introgression of *S. invicta* ancestry into the native *S. richteri* population in Las Flores population, but not in the Buenos Aires population. If hybrids are indeed formed on rare occasions, it may be that such incidents do not lead to detectable gene flow between the species. Alternatively, this may be noise in the *STRUCTURE* analysis. The latter explanation seems more likely because Las Flores is more distant from the *S. invicta* range than Buenos Aires.

A previous study conducted by Ross and Trager (1990) indicated that genetic isolation may be incomplete in the native range [4]. By analyzing six informative allozyme loci (out of 26 that were genotyped) in 100 samples of *S. invicta* and 57 samples of *S. richteri*, three samples were identified as possible hybrids. The results were inconclusive, but occasional admixture between *S. invicta* and *S. richteri* could not be dismissed. Neither could it be determined if the hybrid specimens, assuming it is what they were, are the product of one or many generations of admixture, and so the reproductive potential of native *invicta*-*richteri* hybrids was still unclear. Other studies that used less extensive sampling and more diverse, but still limited, genetic markers of mtDNA sequences and allozyme loci, could identify no signs of admixture [5, 6]. Our conclusion of genetic isolation between these species since they diverged is more powerful than the observation that admixture is not taking place at present time, and is consistent with a strong reproductive barrier. It makes the extensive admixture, observed in North America, even more intriguing. This suggests that the two diverging lineages of *S. invicta* and *S. richteri* were well on their way to complete speciation in South America, but have not reached the point of no return. Their introduction to the USA has resulted in the breakdown of barriers and reversal of this process.

Reproductive isolation between related species is maintained by endogenous (genetic) and/or exogenous (environmental) factors [33]. One explanation for the difference between the admixture patterns in the native and introduced ranges could be that the native populations that were the sources of the introductions are genetically compatible between the species but geographically separated, while the populations whose ranges overlap in South America, are incompatible. This explanation was previously proposed by Ross & Trager (1990) [4]. Since Formosa is located outside of the overlap region, this may well be the case. Another possibility is that the introduced populations have been released from certain exogenous factors, biotic or abiotic, that prevent admixture in the native range. Biotic factors may include parasites that affect mating and reproduction. One such factor is *Wolbachia* – an endosymbiotic bacterium that infects high proportion of insects [34], and manipulates their reproductive systems in ways such as the induction of cytoplasmic incompatibilities between infected and uninfected individuals. Such incompatibilities were found to hinder the admixture between two species of *Drosophila* [35]. As *Wolbachia* was detected in native populations of *S. invicta* and *S. richteri*, but not in the introduced populations [36] it is a strong option that was already pointed out before [6]. Another possibility is changes in the ants’ diet following their introduction to a new environment. Different foods might affect the chemical blend of pheromones the ants produce. In ants and in other insects, social interaction, which includes the identification of a potential sexual mate, relies heavily on chemical cues [37]. In fact, pheromonal differentiation is suspected to be the cause of reproductive isolation between many closely related insect species [38–40]. Moreover, diet was shown to affect the pheromonal profile and sexual desirability in fruit flies [41, 42].

We gave estimates of the number of generations since the two speciation events, which can be converted to years by multiplying them with the average generation time. The social polymorphism of *S. invicta* and *S. richteri* combined with overlapping generations and multiple nuptial flights throughout the year, make the length of a generation highly variable and difficult to pinpoint. Ross and Shoemaker (2008) have given an estimate of three to six years, based on the relative number of alates produced in different developmental stages of a colony [19]. They lean towards the higher end of this range, but maintain that a shorter generation time is more probable for the time following introduction, when their numbers were growing exponentially. Assuming an average generation time of six years, the speciation time between *S. invicta* and *S. richteri* can be estimated to be 1.1 (0.6–2.1) million years ago (MYA) and the speciation time of *S. fugax* and *S. invicta* and *S. richteri* to 15 (9.6–24) MYA. The high end of this estimate is just under the 25 million years (95% confidence interval of 18–32) inferred for the divergence of these lineages, based on Bayesian phylogenetic analysis and divergence dating using 27 fossil calibration points [43]. It should be noted that a phylogeny-based estimate is for the genetic distance between the two representative individuals whose sequences were analyzed, while the coalescent-based-estimate by *3s*, is for the separation of the species (i.e. the formation of genetic isolation). By definition, the divergence between the representative individuals must predate the formation of reproductive barriers that led to speciation.

There is one parameter shared by the two different coalescent-based methods of *DIYABC* and *3s*, which is the native *S. invicta* populations cluster, that of Alejandra and El Recreo. Both methods gave similar estimates for it (2.5*10^5^ and 4*10^5^ respectively). It is approximately two orders of magnitude smaller than *DIYABC* ‘s estimate of 1.2*10^7^ given for the other native population cluster, the Herradura population. This suggests a much larger and more diverse population around Herradura in comparison to the southern population sampled at El Recreo and Alejandra. Of those three locations, Herradura is closest to Corrientes, which was highlighted as the most genetically diverse among the populations that were sampled across South America [44].

The effective population size of the *S. invicta* USA founder population (*N*_*F*_) was estimated at 39. While for diploid species the effective population size is $\mathrm{Ne}=\frac{4\mathrm{NfNm}}{\mathrm{Nf}+\mathrm{Nm}}$ (*N*_*f*_ and *N*_*m*_ are the number of breeding females and males, respectively), for haplodiploid species, the reduced number of chromosomal copies in males results in $\mathrm{Ne}=\frac{4\mathrm{NfNm}}{2\mathrm{Nf}+4\mathrm{Nm}}$ [45, 46]. This translates to a ratio of *N*_*e*_ = 1.5*N*_*f*_ for species in which the queen mates with a single male, as is the case with *S. invicta* [47]. Therefore, the founder population size maximum posterior estimate of 39 translates to 26 unrelated, singly-mated queens, which fits within the higher end of the 9–30 estimate range made by Ross and Shoemaker (2008). If we consider our entire 95% credible interval, then the number of founding *S. invicta* queens is between 10 and 93.

## Conclusion

We described a unique study system in which the reproductive barrier between two closely related species is maintained for approximately 200,000 generations in their native range and breached in an introduced range. This setting allows sampling from clearly separate pure populations in the native range and admixing populations in the introduced range. Therefore, this is a powerful system for the study of the molecular basis of reproductive isolation, which is key to the process of speciation.

## Methods

### Population sampling

Samples were taken from nine ant populations: three *S. invicta* and two *S. richteri* populations in the native range in Northeastern Argentina; two *S. invicta* and two *S. richteri* populations in the introduced range in Southeastern USA. The specimens were identified by morphological characteristics by James Pitts as described before [48]. For each population, 23–51 diploid females were sampled, each from a different colony, totaling 337 individuals. See Fig. 1 and Additional file 1: Tables S1 and S2 for details.

### Sequencing and processing

Seven restriction-site associated DNA (RAD) libraries were constructed from the sampled populations, with 31–68 individuals in each library. DNA was extracted from the samples and RAD libraries were constructed based on the protocols of Baird and colleagues [49] and Etter and colleagues [50], as described by Privman et al. (2018) [27]. Briefly, DNA was digested with PstI-HF enzyme (New England Biolabs) and ligated to one of 96 barcoded P1 adapters with 5 bp unique barcodes. The samples were multiplexed per lane of 100 bp single-end sequencing on an Illumina HiSeq 2000 or 4000 sequencer. A total of 962,896,602 reads, or RADseq, were sequenced, an average of 2,857,259 reads per sample, with the least number of reads for a sample being 197,598 and the most - 6,848,114. Library 3 averaged at the lowest number of reads of 1,628,368 reads per sample. Library 7 averaged at the highest number of reads of 3,335,288 reads per sample. See Additional file 1: Table S1 and S2 for details about samples, reads and sequencing depth.

The raw reads were initially processed using the *Stacks* pipeline [51, 52] and low quality reads were discarded. These were defined as reads in which the phred score drops below 10 (1:10 chance for a sequencing error), averaged over a sliding window of 14 bases. We mapped the reads to 44,102 distinct positions in a *S. invicta* reference genome (version Si_gnH; NCBI accession AEAEQ02000000; [27]) using *Bowtie2* [53]. For each of the mappings, a maximum of two bases mismatch was allowed for its best hit. To maintain the uniqueness of the mapping, we removed alignments with the second best hit containing less than five bases mismatches. Additionally, we controlled for and filtered out reads mapped to what are suspected collapsed repetitive sequences in the reference genome assembly; these were identified based on excessive coverage and heterozygous genotype calls in whole-genome sequencing of 40 haploid *S. invicta* males sampled from Herradura and Alejandra [27].

We analyzed the mapped sequences for single nucleotide polymorphic sites (SNPs) using the *Stacks* pipeline and created a catalog containing 285,847 SNPs for the combined seven RAD libraries, with measured coverage of X16 on average. These SNPs were used to calculate the *F*_*ST*_ distances between the populations.

### Population structure

We filtered the SNPs catalog further before its use in the population structure analyses. For each locus, we required genotype calls in at least 80% of the samples in each population (i.e. less than 20% missing data); a minimum of three reads made for a genotype call. Additionally, we required a minimal minor allele frequency (MAF) of 1%. This stringent filtering resulted in a collection of 16,759 high confidence SNPs. Finally, we removed samples with more than 30% missing data (i.e. loci without genotype calls), leaving 300 samples in the analysis.

We ran *STRUCTURE* over *K* (number of expected clusters) values of 2–9, 100 times for each *K*, with 100 different sets of 1000 SNPs randomly chosen out of the high confidence SNPs collection, a total of 800 separate runs. To avoid linked sites from affecting the analysis, we required a distance of at least 5000 bp between the selected sites. Multiple runs over various data subsets contributed to the robustness of the clustering inference and allowed us to use more SNPs than would be possible in a single run. We ran *STRUCTURE* for 1,100,000 MCMC repetitions and discarded the first 100,000 (burn-in period). All the other parameters were kept at default values. The results were analyzed using *CLUMPAK* [54]. The STRUCTURE analysis was repeated with all the SNPs in the Social chromosome removed, leaving 15,539 SNPs in the analysis. The results were largely unchanged, and they can be found in Additional file 1: Figure S2. We used the Evanno test [55] to identify the number of clusters K, which gave K = 4 (Additional file 1: Figure S3). However, we decided to present the results for K = 5 where the native and introduced *S. richteri* separated to two distinct clusters.

### Populations demographic history

#### Full maximum likelihood test for gene flow using 3s

We tested the hypothesis that *S. invicta* and *S. richteri* have been maintaining complete genetic isolation in South America since their divergence. The alternative to this scenario is that these incipient species have had some form of gene flow between them, including isolated successful mating events (isolation with migration). We used *3s* program, which is a coalescent-based maximum likelihood inference tool. The program analyses aligned sequences of three species, two that are closely related and a third which is used as an out-group, for the genealogical process of coalescence and migration. Based on this the maximum likelihood scores of two possible demographic history models are compared, a null model that does not allow genetic flow between the two closely related species and an alternative model that does.

In addition, *3s* provides estimates for the times of the two speciation events and for historic and contemporary population effective sizes. Speciation times are inferred from the model parameter *τ* – the average number of substitutions per site since speciation. Effective population sizes are represented by the *θ* parameter. In diploid species, *θ* = 4μN_e_, where μ is the mutation rate and *N*_*e*_ is the effective population size. For a haplodiploid species, *θ* = 3*μN*_*e*_ because of the ploidy ratio between males and females.

Using *3s*, we investigated the demographic history of the closely related *S. invicta* and *S. richteri* species with the thief ant *S. fugax* as an out-group (genome assembly version Sf_gnA; NCBI accession QKQZ00000000; [27]). To give meaning to the chosen model parameter estimates of speciation times and effective population sizes, we used the mutation rate of another hymenopteran, the honeybee, which was estimated at 3.4*10^− 9^ (95% confidence interval of 2.2*10^− 9^ - 4.9*10^− 9^) mutations per site per generation. This mutation rate was calculated using direct measurement of two generations in three colonies and is similar to the mutation rate inferred for other insects [56, 57].

##### Genetic data set preparation

The program requires only a small number of individuals with a large number of loci to represent each species. We arbitrarily chose two *S. richteri* individuals from the populations of Las Flores and Buenos Aires (R_1_ and R_2_ respectively) and two *S. invicta* individuals from the populations of Alejandra and El Recreo (I_1_ and I_2_ respectively). All four chosen individuals were of the *gp-9*^*BB*^ genotype of the social chromosome. Using *Bowtie2*, we aligned their RAD reads to the reference genome of *S. invicta* and to a fully sequenced *S. fugax* individual, allowing no gaps in the alignments. We retained only RADseq reads with no more than 4 mismatches compared to the *S. invicta* reference genome and RADseq reads with no more than 10 mismatches compared to the *S. fugax* genome. The mismatches cutoffs are based on the average of 95% sequence identity between *S. invicta* and *S. fugax* [27]. We also required unique mapping and only allowed alignments in which the second best hit has at least twice the number of mismatches as the best hit.

We assembled for each locus an alignment of three sequences composed of the sequences of the outgroup of *S. fugax* and one of following pairs of sequences at random: I_1_ and I_2_, R_1_ and R_2_ or R_1_ and I_2_. As the algorithm assumes no linkage disequilibrium (*LD*) between sites, we only allowed loci that were at least 2000 nucleotides apart. This produced 15,040 triplets of 96 bases long three-ways alignments, to be used as input for *3s* software.

##### Execution

We ran *3s* and calculated the maximum likelihood values for the two models using the Gaussian quadrature number of points = 16. To examine the robustness of the estimates we ran each of the models three times, with different seed values at each run. We used the likelihood scores and the parameter values that were obtained for the highest scoring run of each of the models and compared them in a Likelihood Ratio Test (LRT).

#### Demographic history of populations of *S. invicta*

Using approximate Bayesian computation (ABC), we estimated divergence times and population sizes of the three *S. invicta* population clusters identified by *STRUCTURE*, for the demographic history model depicted in Fig. 4. Instead of full Bayesian calculations of a likelihood function, *DIYABC* uses an approximate approach: it runs a series of coalescent simulations over a demographic history model, which includes an historic scenario and parameter prior distributions. The program estimates the posterior distributions for the model parameters based on how well the simulated data fit the observed genetic data (represented by their summary statistics).

Our *STRUCTURE* analysis found the two native *S. invicta* populations of Alejandra and El Recreo indistinguishable in term of their genetic polymorphism and so they were assigned to one population, while Herradura’s population was clustered separately. The introduced populations were also clustered together in one population. We formulated the demographic history model used for the ABC analysis with these three distinct population clusters.

##### Scenario construction

In the historic scenario, depicted in Fig. 4, the two native populations, *N*_*N1*_ and *N*_*N2*_, diverged from each other *T*_*D*_ generations ago, prior to the introduction to the USA, which took place *T*_*I*_ generations ago. As the introduced populations in the USA were found to originate from the region of Formosa (which includes Herradura), in our scenario the founder population, *N*_*F*_, is directly derived from population *N*_*N1*_, and their divergence time coincides with the time of introduction. The scenario also includes a population bottleneck effect after the introduction, which lasted for *T*_*B*_ generations and concluded as the size of the population reached its contemporary size, *N*_*I*._

##### Parameterization

Coalescent model parameters are either demographic, reflecting population effective sizes, or temporal, indicating the number of generations that had passed between events. We defined parameter priors of normal distributions with averages based on known life history of the fire ants. Prior distribution widths were set widely to allow *DIYABC* sizeable sampling range, encompassing all reasonable values of these parameters.

The introduction time of *S. invicta* predates sampling time by at least 80 years. Generation time for the fire ants, especially at the time they were dispersing throughout the newly introduced range, is hard to estimate. We therefore defined a prior of introduction time that ranges between 0 to 180 generations. Based on studies indicating that the original number of introduced *S. invicta* was small, we defined a prior for the effective population size that ranges between 1 and 500 individuals. The ants’ quick expansion in North America suggests a minimal time span of the population bottleneck. This allowed us to narrow the prior distribution of the bottleneck time between 0 and 40 generations. Not much is known about the effective sizes of the native and introduced populations or of the time of divergence between the native populations, and these parameter priors were kept at a wide distribution, which included many orders of magnitudes.

##### Choice of summary statistics

An ABC analysis depends on population summary statistics to reduce the high-dimensional genetic information and to evaluate the simulation results. A varied choice of summary statistics is therefore crucial. However, using too many would increase the dimensionality of the analysis, making the available observed data points too sparse in comparison [58]; a problem known as “the curse of dimensionality”.

The native *N*_*N1*_ and *N*_*N2*_ populations are presumed stable and well represented by their summary statistics. We therefore used all of the within-population summary statistics offered by *DIYABC*: the proportion of monomorphic alleles, mean and variance of gene diversity of polymorphic loci [59] and mean gene diversity across all loci. For the population *N*_*I*_, a newly established population, we only used the summary statistics of the proportion of monomorphic alleles and mean gene diversity across all loci.

We included between-populations summary statistics for pairs of populations that directly diverged from each other (*N*_*N1*_ & *N*_*N2*_ and *N*_*N1*_ & *N*_*I*_): the mean *F*_*ST*_ distance, the proportion of null *F*_*ST*_ distances [60], and mean and variance of non-null *F*_*ST*_ distances.

##### Genetic data set preparation

We subdivided the SNPs catalog to create a dataset to include only the *S. invicta* samples. The SNPs were filtered as described before, with the following modifications: we required that each locus has genotype calls in at least 90% of the samples of each population, a minimal MAF of 0.5%, and at least 5000 bp between SNPs. The reduced MAF threshold is meant to allow the *DIYABC* analysis to make use of the valuable information in the low frequency alleles. This resulted in 161 samples and 6389 loci that were used in the analysis.

##### Simulations and analyses

Running *DIYABC* coalescent simulations, we created 892,000 simulated data sets. Based on the 1% of the simulations that produced summary statistics closest to the observed data, the parameter posterior distributions were estimated and adjusted using a weighted linear regression in which the summary statistics were the independent variables. To measure the goodness of fit of the model [61], we randomly selected 10% of the adjusted simulated data sets, and compared them to the observed data in a PCA analysis of the summary statistics.

## Additional file

Additional file 1: **Table S1.** Populations sampling times, locations, numbers and social form genotypes. Also detailed is the average coverage per ant for each of the population after the completion of initial filtering to the point of unique mapping to the reference genome. **Table S2.** RAD libraries composite and raw reads number. **Figure S1.** *STRUCTURE*’s major run results for K = 2–9. **Figure S2.** *STRUCTURE*’s major run results for K = 2–9 with SNPs linked to chr16 (social chromosome) removed, leaving 15,539 SNPs in the analysis. Populations as before. **Figure S3.** Evanno analysis for best K. **Table S3.** - *F*_*ST*_ between populations. **Table S4.** Posterior distributions of *S. invicta* demographic history model parameters. **Figure S4.** Goodness of fit analysis result for *S. invicta* demographic history model. (DOCX 446 kb)

## Abbreviations

ABC: Approximate Bayesian computation
CSD: Sex determination locus
LD: Linkage disequilibrium
MAF: Minor allele frequency
MYA: Million years ago
RAD: Restricted site associated DNA
RADseq: RAD sequence
SNP: Single nucleotide polymorphism
