# Supplementary material for: Speciation and hybridization in invasive fire ants
Source: BMC Evol Biol. 2019 May 29;19:111. doi: 10.1186/s12862-019-1437-9 (PMC6542140; doi:10.1186/s12862-019-1437-9)
Supplement: Supplementary file 1 — Table S1. Populations sampling times, locations, numbers and social form genotypes. Also detailed is the average coverage per ant for each of the population after the completion of initial filtering to the point of unique mapping to the reference genome. Table S2. RAD libraries composite and raw reads number. Figure S1. STRUCTURE’s major run results for K = 2–9. Figure S2. STRUCTURE’s major run results for K = 2–9 with SNPs linked to chr16 (social chromosome) removed, leaving 15,539 SNPs in the analysis. Populations as before. Figure S3. Evanno analysis for best K. Table S3. - FST between populations. Table S4. Posterior distributions of S. invicta demographic history model parameters. Figure S4. Goodness of fit analysis result for S. invicta demographic history model. (DOCX 446 kb) [file 12862_2019_1437_MOESM1_ESM.docx]

| Species | Range | Collectors  Collection year | Location | Samples | Coverage | BB/Bb/bb social form |
| --- | --- | --- | --- | --- | --- | --- |
| *S. invicta* | Native | Luis Calcaterra  2006 | Herradura, Formosa, Argentina (S26°29.826', W58°18.950')  Horse Club pasture | 31 gynes | X27.02  (18.6-38.3) | 31/0/0 |
|  |  | Luis Calcaterra  2006 | El Recreo, Santa Fe, Argentina (S31º30.303', W60º44.455')  El Recreo, pasture | 30 workers | X8.54  (0.17 – 14.45) | 28/2/0 |
|  |  | Luis Calcaterra  2006 | Alejandra, Santa Fe, Argentina (S29º49.543', W59º48.602')  Route-side in route 1 | 38  workers | X9.22  (2.7 – 18) | 29/9/0 |
|  | Introdu-ced | D. DeWayne Shoemaker 2009 | Pascagoula, Mississippi, USA (N30º24', W88º31')  MS- Hwy 63 between MLK Bdvd and shortcut road on the exit side (north bound 63); west side of HWY 63, southbound, between Federick St and shortcut road; west side of HWY 63, between shortcut road and HWY 90 | 46 gynes | X11.85  (0 – 28.5) | 36/10/0 |
|  |  | Kenneth G. Ross  2011 | Oglethorpe Co., Georgia, USA (N33º56', W83º23') | 48 gynes | X20.71  (0.25 – 44)) | 47/1/0 |
| *S. richteri* | Native | Andy Bouwma  Luis Calcaterra  2005 | Buenos Aires, Argentina  (S34º45.096', W59º10.501')  Estancia, San Mariano | 26 workers | X14.57  (6.3 – 23.5) | 4/18/4 |
|  |  | Andy Bouwma  Luis Calcaterra  2006 | Las Flores, Argentina  (S36º02.881', W59º05.382')  Las Flores | 46 workers | X18.32  (0.165 – 43.8) | 42/4/0 |
|  | Introdu-ced | Kenneth G. Ross  2011 | Benton Co., Tennessee, USA  (N35º51.49', W88º05.998')  Near park of Holladay;  Along US 641 within 3 miles north of junction with I40 | 49 gynes | X17.75  (1.27 – 30.6) | 49/0/0 |
|  |  | D. DeWayne Shoemaker 2005 | Flatwood, Tennessee, USA  (N35º50.37', W88º05.9761')  I40 at exit 126;  Flatwood Baptist Church | 23 workers | X14.65  (0.11 – 24.9) | 23/0/0 |

Table S1. Populations sampling times, locations, numbers and social form genotypes. Also detailed is the average coverage per ant for each of the population after the completion of initial filtering to the point of unique mapping to the reference genome.

Table S2. RAD libraries composite and raw reads number.

| Library | Populations | Raw reads |
| --- | --- | --- |
| Herr | *S. invicta* from Herradura , Argentina | 130,545,860 |
| 2 | *S. richteri* from Buenos Aires, Argentina  *S. richteri* from Flatwood, Tennessee | 145,259,449 |
| 3 | *S. invicta* from El Recreo, Argentina  *S. invicta* from Alejandra, Argentina | 110,729,059 |
| 4 | *S. invicta* from Pascagoula, MS | 100,738,579 |
| 5 | *S. richteri* from Las Flores, Argentina | 159,778,609 |
| 6 | *S. richteri* from Benton Co., TN | 155,751,198 |
| 7 | *S. invicta* from Oglethorpe Co., GA | 160,093,848 |


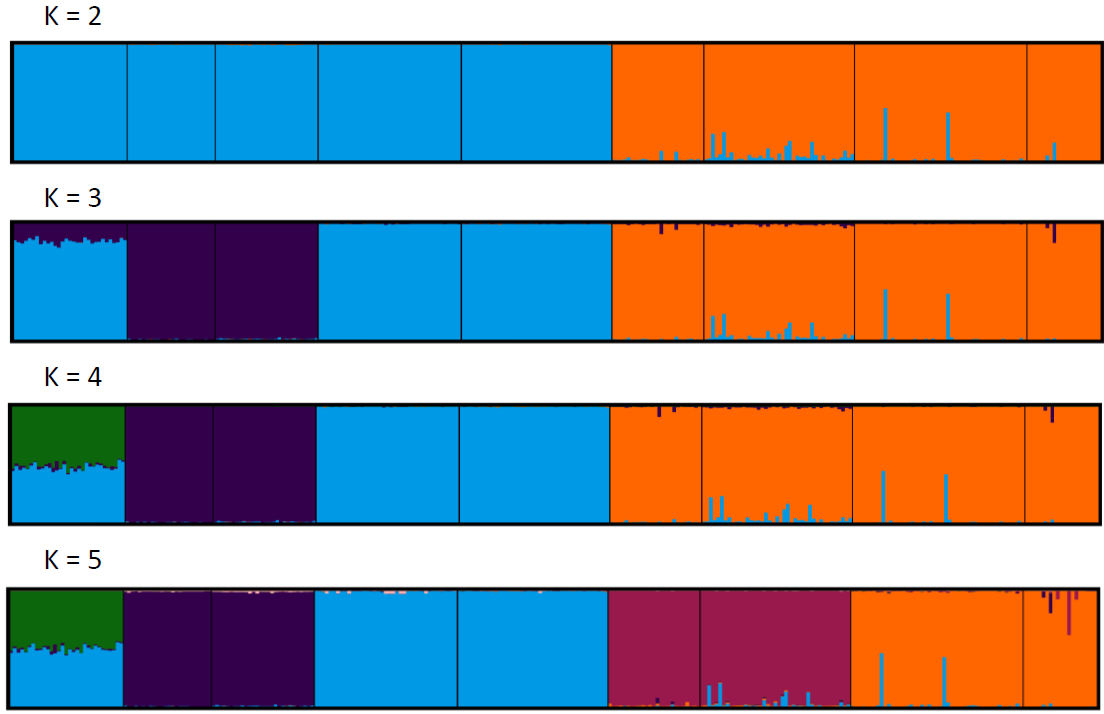


­
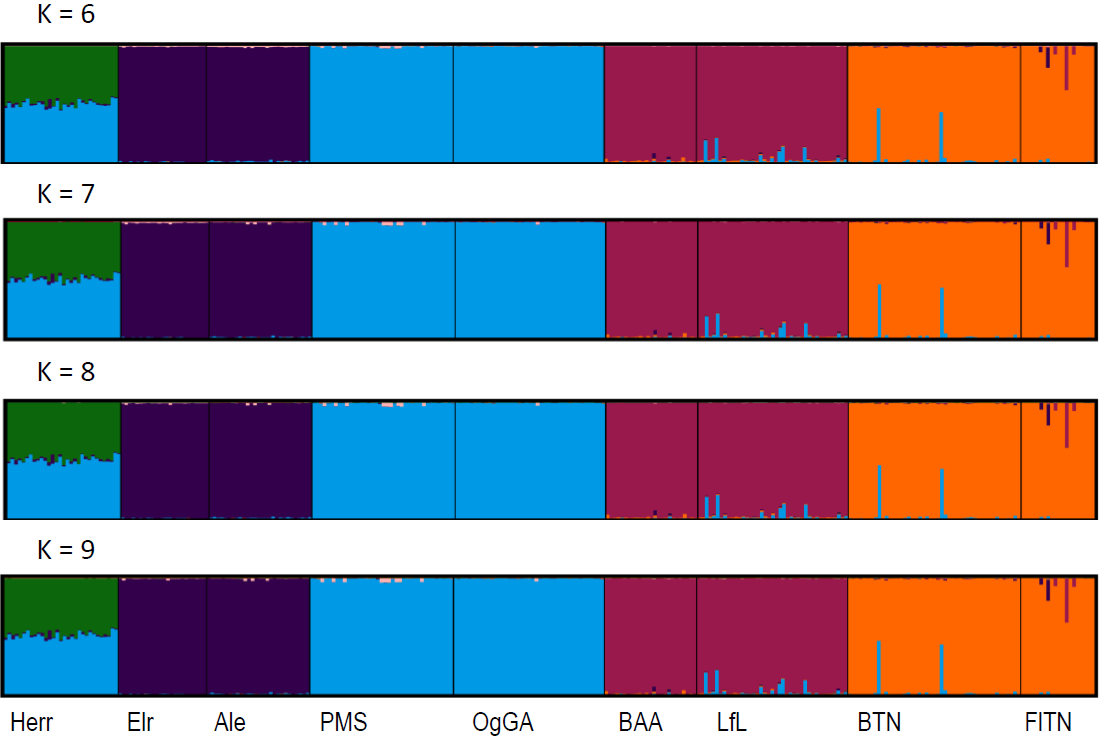


**Figure S1.** *STRUCTURE*’s major run results for K = 2 – 9. Populations are: *Herr* - Herradura, *Elr* – El Recreo, *Ale* – Alejandra, *PMS* - Pascagoula, Mississippi, *OgGa* - Oglethorpe Co., Georgia, *BAA* - Buenos Aires, *LfL* - Las Flores, *BTN* - Benton Co., Tennessee *FlTN* - Flatwood, Tennessee.


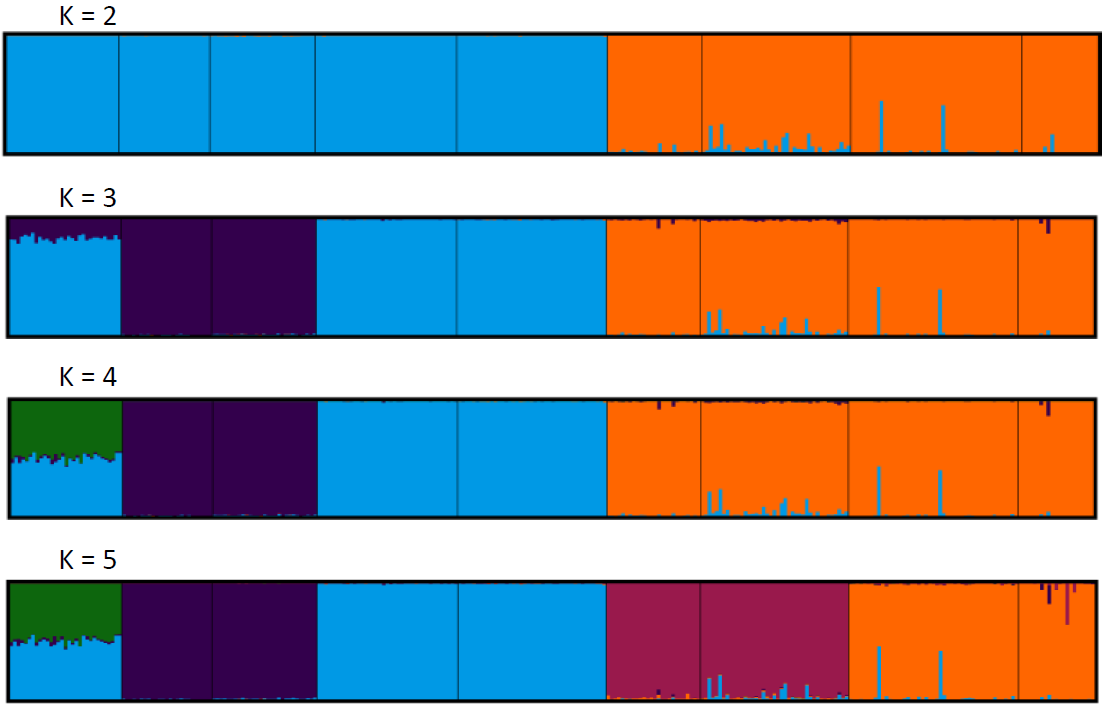


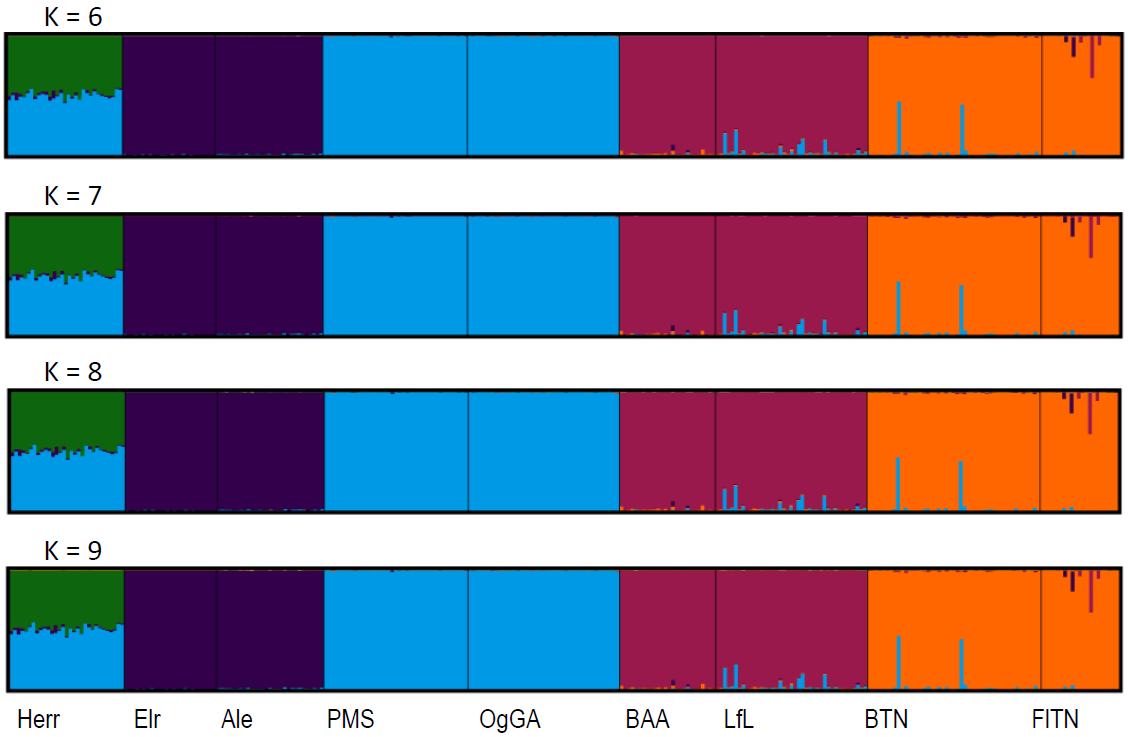


**Figure S2**. *STRUCTURE*’s major run results for K = 2 – 9 with SNPs linked to chr16 (social chromosome) removed, leaving 15,539 SNPs in the analysis. Populations as before.


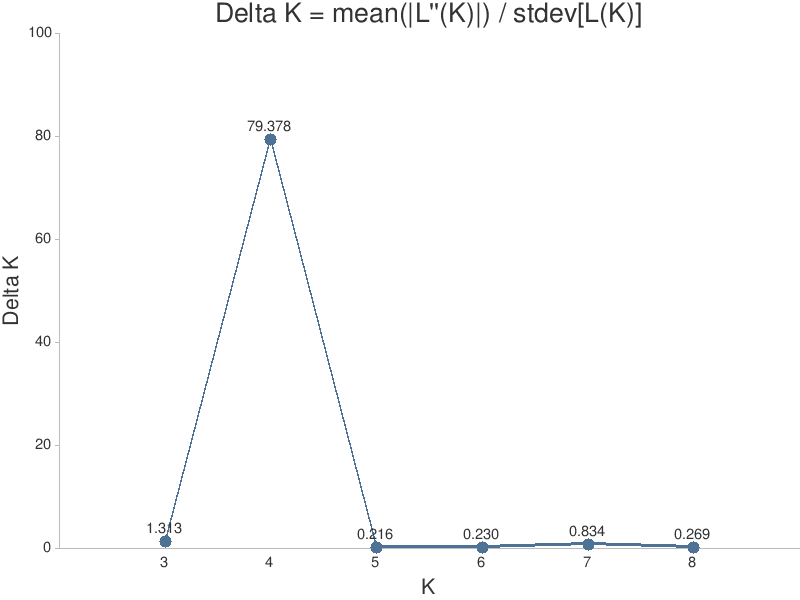


**Figure S3**. Evanno analysis for best K.

Table S3 - *F_ST_* between populations. *Sinv – S. invicta, Srich – Solenopsis richteri*. *Herr* - Herradura, *Elr* – El Recreo, *Ale* – Alejandra, *PMS* - Pascagoula, Mississippi, *OgGA* - Oglethorpe Co., Georgia, *BAA* - Buenos Aires, *LfL* - Las Flores, *BTN* - Benton Co., Tennessee *FlTN* - Flatwood, Tennessee.

|  | SinvHer | SinvElR | SinvAle | SinvPMS | SinvOgGA | SrichBAA | SrichLfL | SrichBTN | SrichFlTN |
| --- | --- | --- | --- | --- | --- | --- | --- | --- | --- |
| SinvHer |  | 0.043376 | 0.042152 | 0.034781 | 0.041544 | 0.107648 | 0.092859 | 0.135648 | 0.1292 |
| SinvElR |  |  | 0.011071 | 0.057511 | 0.078674 | 0.149662 | 0.117093 | 0.210294 | 0.199363 |
| SinvAle |  |  |  | 0.049589 | 0.065835 | 0.130656 | 0.106053 | 0.179606 | 0.167832 |
| SinvPMS |  |  |  |  | 0.009239 | 0.155797 | 0.123105 | 0.211027 | 0.19917 |
| SinvOgGA |  |  |  |  |  | 0.196189 | 0.156357 | 0.282353 | 0.262383 |
| SrichBAA |  |  |  |  |  |  | 0.011814 | 0.071175 | 0.055141 |
| SrichLfL |  |  |  |  |  |  |  | 0.053248 | 0.042019 |
| SrichBTN |  |  |  |  |  |  |  |  | 0.015338 |

**Table S4.** Posterior distributions of *S. invicta* demographic history model parameters. In red are the maximum posterior estimates. Populations: *N_N1_* – native population of Herradura; *N_N2_* – native population of Alejandra and El Recreo; *N_F_* – bottlenecked founder population in the USA; *N_I_* – contemporary population in the USA. Times: 0 – populations sampling; *T_I_* – introduction; *T_B_* – length of bottleneck for the introduced population; *T_D_* - divergence of the Herradura population from the Alejandra and El Recreo populations.

| Parameter | Mean | Median | Mode | Q025 | Q050 | Q250 | Q750 | Q950 | Q975 |
| --- | --- | --- | --- | --- | --- | --- | --- | --- | --- |
| N_N1_ | 2.12e+07 | 1.72e+07 | 1.17e+07 | 3.28e+06 | 4.73e+06 | 1.12e+07 | 2.49e+07 | 5.23e+07 | 7.76e+07 |
| N_N2_ | 5.07e+05 | 3.41e+05 | 2.48e+05 | 8.20e+04 | 1.03e+05 | 2.15e+05 | 5.73e+05 | 1.46e+06 | 1.98e+06 |
| N_I_ | 1.42e+06 | 1.25e+06 | 9.41e+05 | 6.80e+04 | 1.33e+05 | 6.40e+05 | 2.03e+06 | 3.27e+06 | 3.67e+06 |
| T_I_ | 4.66e+01 | 4.37e+01 | 3.56e+01 | 9.48e+00 | 1.28e+01 | 2.79e+01 | 6.20e+01 | 9.06e+01 | 9.94e+01 |
| T_B_ | 3.95e+00 | 2.79e+00 | 1.96e+00 | 0 | 5.32e-01 | 1.65e+00 | 4.72e+00 | 1.22e+01 | 1.57e+01 |
| N_F_ | 5.60e+01 | 4.93e+01 | 3.91e+01 | 1.36e+01 | 1.76e+01 | 3.38e+01 | 7.05e+01 | 1.17e+02 | 1.39e+02 |
| T_D_ | 1.28e+05 | 1.13e+05 | 8.20e+04 | 2.32e+04 | 3.21e+04 | 7.25e+04 | 1.68e+05 | 2.78e+05 | 3.20e+05 |

**
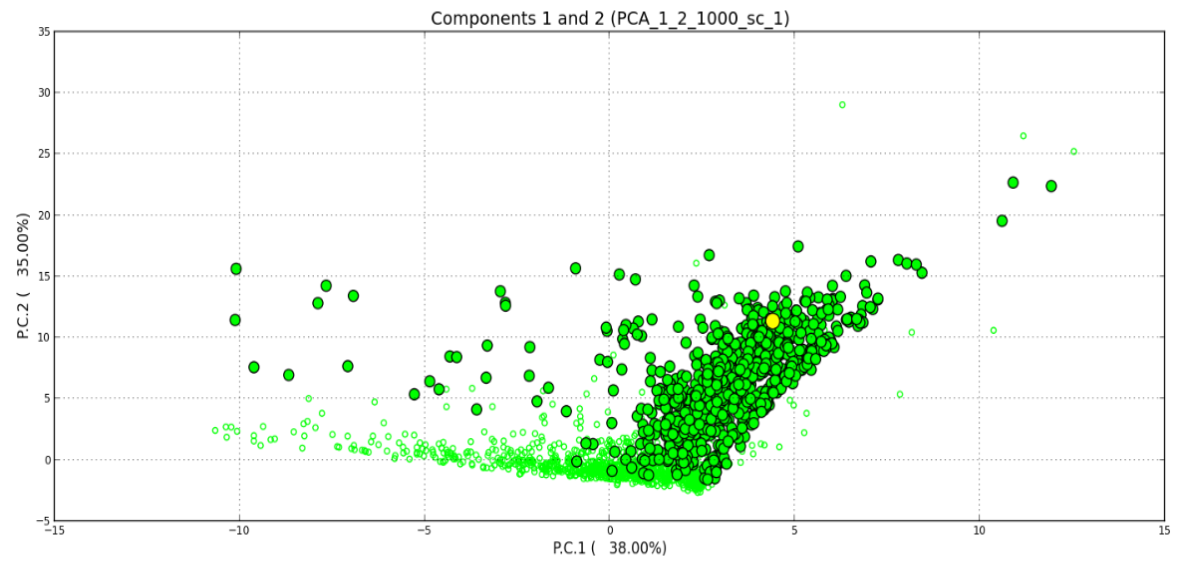
**

**Figure S4.** Goodness of fit analysis result for *S. invicta* demographic history model. The empty green dots represent the selected prior of the parameters; the full green dots, the calculated posteriors; the yellow dot is the observed data set.
